# A likely case of progression from mild cognitive impairment to dementia in Yasunari Kawabata's *The Sound of the Mountain*

**DOI:** 10.1590/0004-282X-ANP-2021-0373

**Published:** 2022-02-28

**Authors:** Matheus Kahakura Franco Pedro, Amanda Batista Machado

**Affiliations:** 1 Instituto de Neurologia de Curitiba, Departamento de Neurologia, Curitiba PR, Brazil. Instituto de Neurologia de Curitiba Departamento de Neurologia Curitiba PR Brazil; 2 Instituto de Neurologia de Curitiba, Departamento de Neurocirurgia Endovascular e Neurorradiologia Intervencionista, Curitiba PR, Brazil. Instituto de Neurologia de Curitiba Departamento de Neurocirurgia Endovascular e Neurorradiologia Intervencionista Curitiba PR Brazil

**Keywords:** Medicine in Literature, Cognitive Dysfunction, Dementia, Aging, Medicina na Literatura, Disfunção Cognitiva, Demência, Envelhecimento

## Abstract

Ageing has always been a prominent theme for many authors, who wrote about the physical and cognitive changes that accompany it. Japanese literature, in particular, is rich in examples, especially from the pen of Yasunari Kawabata. In *The Sound of the Mountain*, Kawabata narrates the old age of Shingo Ogata, who begins the book manifesting only lapses in episodic memory, in a manner compatible with what we would call mild cognitive impairment. After detailed descriptions of other ailments of old age, Shingo comes to realise that a new deficit has appeared: apraxia. Unable to tie his own tie, he realises his own decline to what we could call an initial form of dementia, with this added cognitive deficit impacting his daily life. In short, Kawabata elegantly delineates a disease progression familiar to all neurologists, in a way that leads us to consider with new lenses the neurological challenges of ageing.

## RESUMO

O envelhecimento sempre foi um tema de proeminência para diversos autores, que escreveram sobre as mudanças físicas e cognitivas que o acompanham. A literatura japonesa, particularmente, é rica em exemplos, como Yasunari Kawabata. Em *O Som da Montanha*, Kawabata narra a vida de Shingo Ogata que, no começo, manifesta apenas sintomas em memória episódica, preenchendo critérios diagnósticos para o que hoje chamamos de comprometimento cognitivo leve. Após descrições detalhadas de outros problemas de saúde relacionados à idade, Shingo percebe que outro sintoma se manifestou: apraxia. Incapaz de dar nó em sua gravata, ele percebe seu próprio declínio para o que hoje chamamos de demência, já que este novo déficit passa a ter impacto em sua funcionalidade. Em resumo, Kawabata elegantemente descreve uma progressão de doença familiar a todos os neurologistas, usando uma voz poética que nos faz enxergar com novas lentes os desafios neurológicos do envelhecimento.

## INTRODUCTION

 The demographic composition of Japan has led the country to a singular status, in which the elderly population (citizens above 65 years of age) has become an ever more prominent group in the last few decades^1^; declining mortality rates and changes in fertility and family structures contributed to a population of elderly in excess of 25% of the total population^2^. Given the impact of this ageing in different aspects of contemporary Japan, such as social security, employment, and political scenery, it is natural that the culture would be impacted as well^3^.

 Age and ageing have been a recurrent theme in Japanese literature^4^; traditionally, respect is given to the elders, often seen as wise sages, with religious undertones, either from Shinto and from Buddhism^5^. This venerable, if somewhat overly idealised figure, respected by all in quasi-mystical fashion^6^, finds literary representatives in masters such as Oyake no Yotsugi and Natsuyama no Shigeki in the anonymous *Okagami* tale, from circa 1119^7^, and, more recently, Kenpo Yoshioka, made popular in *Musashi*, by Eiji Yoshikawa^8^. In the past few decades, the rapid population shift and the aforementioned changes led to a more cynical view of old age: Jun'ichiro Tanizaki's Utsugi Tokosuge, in *Diary of an Old Man* (*Futen rojin nikki*), a book from 1961, is himself a neurological patient, victim of a stroke that left him with an upper limb monoplegia^9^; Shusaku Endo's *Scandal* (*Sukyandaru*), from 1986, follows Suguro, confronted by ailing body and cognition, the death of similarly aged friends, and a dostoyevskian *doppelgänger* which mirrors his increasing physical incapability^10^; Kenzaburo Oe's quasi autobiographical Cogito Shoko is the protagonist of a series of novels, like *Death by Water* (*Suichi*), from 2009, in which the author unravels ageing and the social changes that accompany it^11^; however, no author surpassed Yasunari Kawabata in depicting ageing and its neurological ailments in contemporary Japan.

### Yasunari Kawabata and *The Sound of the Mountain*

**Figure 1.**
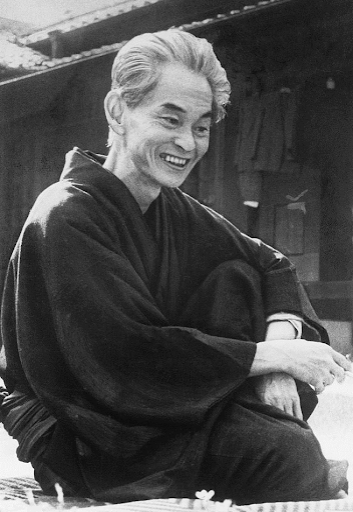
Portrait of Yasunari Kawabata, circa 1968.

Source: Wikimedia Commons.

 Yasunari Kawabata (1899-1972) was a novelist from Osaka (Figure 1); he studied English and Japanese Literature at the Tokyo Imperial University (now University of Tokyo)^12^. Shortly after graduating, he published *The Dancing Girl of Izu* (*Izu no odoriko*)^13^, in 1926, followed by *Snow Country* (*Yukiguni*)^14^, serialised from 1935 to 1937. Other major novels include *Thousand Cranes* (*Sembazuru*)^15^, from 1949 to 1951, and *Kyoto*^16^, from 1962. His career culminated in the first ever Japanese Nobel Prize for Literature, in 1968. He passed away in 1972, in an apparent suicide, possibly motivated by sadness over the loss of lifetime friend and fellow writer Yukio Mishima, who had greatly influenced his own writing^17^, and realisation of the severity of his own illness (idiopathic Parkinson's disease)^12^. However, his popularity and relevance have not waned after his death, and he has been widely translated, with current editions in Portuguese published by Estação Literária.

 Ageing is a prominent motif in Kawabata's *oeuvre*: *The Master of Go* (*Meijin*), published in 1951, depicts Honinbo Shisai, the titular elderly master, who, in a manner reminiscent of Stefan Zweig, faces and is ultimately defeated by a much younger opponent, losing his life purpose^18^; in *The House of the Sleeping Beauties* (*Nemureru bijo*), Old Eguchi is haunted by his own physical and moral decay^19^.

 It is in *The Sound of the Mountain* (*Yama no Oto*)^20^, though, that Kawabata's prowess in depicting the intersection of ageing and an acute description of neurological illness is crucially revealed. Serialised from 1949 to 1954, it is the story of Shingo Ogata, a 62-year-old male, nearing retirement, who finds himself increasingly aware of his waning years, especially when contrasted to his slightly older wife, Yasuko : “Shingo could not tell the age at which she started to look the younger of the couple. (...) Women generally age faster than men, but, in their case, the opposite has happened.” (p. 13-14, our translation).

 Neurologically, the story begins with Shingo manifesting symptoms compatible with mild cognitive impairment, with occasional lapses in episodic memory, limited to this single cognitive domain and without any interference in his daily activities; the author describes Shingo forgetting the name of their recently fired maid: “Five days ago? Just five days ago she left and I can’t remember anything about her… (...) I can’t even think of her name.” (p. 12, our translation).

Kawabata describes recurrent dreams of deceased friends, as well as a sound Shingo believes that comes from the nearby mountain; he interprets both as bad omens. Though Shingo strived his entire life to maintain an orderly life, he sees the morals of his family crumble as his son becomes an adulterer and his daughter abandons her husband and he is left to minimise the fallout. His repeated failings in keeping his family on the right path take a toll on him, and he realises the worsening of his own cognition in climatic fashion as he develops apraxia and fails to knot his necktie, which had done flawlessly, daily for over 40 years:

“[He] felt his hands go wrong. He untied and tried again, but had no more success in his second attempt. (...) Why would have he suddenly forgotten a process he had repeated every morning during a career of forty years? His hands should move automatically; he should have tied his tie without even thinking.” (p. 313-317, our translation).

Now, with a second cognitive domain affected and interference in his daily activities, it is plausible to presume he has progressed to an actual dementia, even if still in an early, mild phase.

The author also provides striking descriptions of presbyopia and presbyacusia:

“Without glasses, he had difficulties differentiating the hour hand from the minute hand [on his watch].” (p. 179, our translation)

“The temple bells rang all day long; there were occasions when Shingo could not hear them. Kikuko could, even as she worked and talked, but Shingo had to listen with care”. (p. 203, our translation)

In conclusion, ageing is a universal phenomenon. In Japan, due to demographic peculiarities, it has become a culturally ingrained element, with multiple literary descriptions. Specifically, mild cognitive impairment and dementia, common in old age, are exquisitely described in Yasunari Kawabata’s arguable magnum opus, fulfilling what we use today as diagnostic criteria, thus highlighting the relevance of neurological diseases in the cultural repertory and imaginarium of literature's greats. Literature allows for a vast range of dialogues and exchanges with the medical arts; the analysis of medical entities in works of art constitute one of these dialogues, in which the disease serves not as a path of limiting oversimplification or stereotyping of a character based on an ailment, but as a tool to see a literary perspective on health and disease, as if through new lenses; thus, the author deeply humanises the character and provides an outsider, refreshing perspective on the topics of medical study.
